# Upregulation of mesothelial genes in ovarian carcinoma cells is associated with an unfavorable clinical outcome and the promotion of cancer cell adhesion

**DOI:** 10.1002/1878-0261.12749

**Published:** 2020-06-25

**Authors:** Kaire Ojasalu, Corinna Brehm, Kristin Hartung, Maximilian Nischak, Florian Finkernagel, Peter Rexin, Andrea Nist, Evangelos Pavlakis, Thorsten Stiewe, Julia M. Jansen, Uwe Wagner, Stefan Gattenlöhner, Andreas Bräuninger, Sabine Müller‐Brüsselbach, Silke Reinartz, Rolf Müller

**Affiliations:** ^1^ Center for Tumor Biology and Immunology Philipps University Marburg Germany; ^2^ Institute of Pathology Philipps University Marburg Germany; ^3^ Institute of Pathology Justus‐Liebig University Giessen Germany; ^4^ Genomics Core Facility Philipps University Marburg Germany; ^5^ Institute of Molecular Oncology Member of the German Center of Lung Research (DZL) Philipps University Marburg Germany; ^6^ Clinic for Gynecology Gynecological Oncology and Gynecological Endocrinology University Hospital Giessen and Marburg (UKGM) Marburg Germany; ^7^ Present address: Institute of Pathology Aurich and Ammerland Aurich Germany

**Keywords:** adhesion, calretinin, driver mutations, mesothelial cells, metastasis, ovarian cancer, podoplanin, transcriptomics

## Abstract

A hallmark of ovarian high‐grade serous carcinoma (HGSC) is its early and massive peritoneal dissemination via the peritoneal fluid. It is generally believed that tumor cells must breach the mesothelium of peritoneal organs to adhere to the underlying extracellular matrix (ECM) and initiate metastatic growth. However, the molecular mechanisms underlying these processes are only partially understood. Here, we have analyzed 52 matched samples of spheroids and solid tumor masses (suspected primary lesions and metastases) from 10 patients by targeted sequencing of 21 loci previously proposed as targets of HGSC driver mutations. This analysis revealed very similar patterns of genetic alterations in all samples. One exception was *FAT3* with a strong enrichment of mutations in metastases compared with presumed primary lesions in two cases. *FAT3* is a putative tumor suppressor gene that codes for an atypical cadherin, pointing a potential role in peritoneal dissemination in a subgroup of HGSC patients. By contrast, transcriptome data revealed clear and consistent differences between tumor cell spheroids from ascites and metastatic lesions, which were mirrored by the *in vitro* adherence of ascites‐derived spheroids. The adhesion‐induced transcriptional alterations in metastases and adherent cells resembled epithelial–mesenchymal transition, but surprisingly also included the upregulation of a specific subset of mesothelial genes, such as calretinin *(CALB2)* and podoplanin *(PDPN)*. Consistent with this finding, calretinin staining was also observed in subsets of tumor cells in HGSC metastases, particularly at the invasive tumor edges. Intriguingly, a high expression of either *CALB2* or *PDPN* was strongly associated with a poor clinical outcome. siRNA‐mediated *CALB2* silencing triggered the detachment of adherent HGSC cells *in vitro* and inhibited the adhesion of detached HGSC cells to collagen type I. Our data suggest that the acquisition of a mesenchymal–mesothelial phenotype contributes to cancer cell adhesion to the ECM of peritoneal organs and HGSC progression.

## Graphical abstract

Attachment of ovarian cancer cells to damaged mesothelium is a critical step in metastatic spread. This study suggests that cancer cell adhesion to the extracellular matrix (ECM) is linked to mutations in the atypical cadherin and tumor suppressor *FAT3* in a subset of patients, as well as to a mesenchymal–mesothelial phenotype involving the upregulation of calretinin and podoplanin.

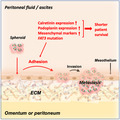

**Abbreviations**
CDS: coding sequence
ECM: extracellular matrix;
EMT: epithelial–mesenchymal transition
FBS: fetal bovine serum
GO: gene ontology
HGSC: high‐grade serous carcinoma
INV: inversion
NGS: next‐generation sequencing
OC: ovarian cancer
OCMI medium: Ovarian Carcinoma Modified Ince medium
OS: overall survival
RNA‐Seq: RNA sequencing
qRT‐PCR: quantitative reverse transcriptase–PCR
RFS: relapse‐free survival
SNV: single nucleotide variant
SV: structural variants
TAM: tumor‐associated macrophage
TAT: tumor‐associated T cell
TCGA: The Cancer Genome Atlas

## 1 Introduction

Ovarian carcinoma (OC) is the deadliest of all gynecological malignancies with high‐grade serous carcinoma (HGSC) as the most common subtype. Typically, HGSC is diagnosed at advanced stages with metastases in the abdomen beyond the pelvis. HGSOC is characterized by a very high frequency of *TP53* and BRCA1/2 mutations (approximately 97% and 40%, respectively), as well as amplification and overexpression of *MYC* (>50%) [1]. HGSC is thought to arise from the fimbriated fallopian tube epithelium [2]. Serous tubal intraepithelial carcinomas may represent potential precursor lesions, although this issue remains controversial [3].

A hallmark of HGSC is its tumor microenvironment, which is composed of anatomically and functionally different compartments [4]. These include the solid tumor masses invading host tissues (most notably the omentum) and the peritoneal fluid, which often occurs as ascites at advanced stages. This malignancy‐associated peritoneal fluid contributes to the fatal nature of HGSC by enabling peritoneal dissemination. Tumor cells are shed at a very early stage of the disease, and can be detected in lavaged peritoneal fluid even at a stage when the tumor is still confined to the oviduct or ovary [5]. Due to its active role in peritoneal dissemination, HGSC ascites is unique compared with other human cancers, where effusions are reactive or occur as a secondary phenomenon. The essential role of transcoelomic metastatic spread in HGSC is further pointed by the observation that, in contrast to most other cancers, metastases at distant sites are confined to very late stages [5]. The most serious problem for most HGSOC patients is therefore the dissemination and aggressive growth of metastatic lesions within the peritoneal cavity.

Ascites‐associated cancer cells occur as single cells or multicellular spheroids, thought to be responsible for peritoneal dissemination [6]. Besides these tumor cells, tumor‐associated macrophages and T cells are the most common cell types in ovarian carcinoma‐associated ascites [7, 8, 9]. Soluble factors and extracellular vesicles released by these cells provide an environment that strongly supports tumor progression, chemoresistance, and immune evasion [4, 10, 11, 12, 13, 14, 15, 16, 17].

Although the vast majority of ovarian carcinomas are highly responsive to chemotherapy, most patients suffer from recurrence within 3 years [18]. In contrast to the therapy resistance of relapsed tumors in other entities, many relapsed HGSC cases are still highly sensitive to treatment. This could be explained by the toggling of cancer cells between states of transient chemosensitivity and chemoresistance mediated by changes in the tumor microenvironment. Thus, a small number of transiently resistant tumor cells survive first‐line chemotherapy and later on, due to their high tumorigenic potential, initiate new metastatic lesions upon attachment to visceral organs [19, 20].

The metastatic spread of ovarian cancer cells depends on the invasion into the mesothelium covering the peritoneal organs. The mesothelium consists of a single layer of mesothelial cells covering a basement membrane composed of the extracellular matrix (ECM) proteins collagen, fibronectin, and laminin. It has been reported that OC cells can attach directly to mesothelial cells via β1 integrin [21, 22, 23] or CD44 [22, 24, 25], concomitantly with the upregulation of genes linked to epithelial–mesenchymal transition (EMT) and the downregulation of epithelial genes [26]. However, the relevance of these cancer–mesothelial cell interactions in the context of OC cell invasion remains unclear.

The mesothelium is rather believed to act as a barrier and therefore as first line of defense against invasion by cancer cells in the peritoneal fluid [20]. This is supported by the observation that metastatic sites are typically devoid of mesothelial cells [27, 28]. It has been hypothesized that OC cells attach to the ECM at preexisting lesions of the mesothelial cell layer, which might occur spontaneously and/or could be triggered by changes in cell morphology due to mesothelial cell activation by components of the malignancy‐associated peritoneal fluid [29, 30], by the induction of mesothelial cell senescence [31], by myosin‐dependent mechanical forces exerted by tumor cells [32], or by mesothelial cell killing by FAS ligand [33]. After passing through gaps in the mesothelial cell layer, cells adhere to, and disrupt, the submesothelial basement membrane to invade into the underlying tissue [27]. Consistent with these findings, OC cells adhere strongly to basement membrane proteins coated onto culture dishes, whereas there is only weak attachment, if any, to mesothelial cells [28].

In spite of the knowledge summarized above, HGSC attachment as a first step to metastasis formation is only partially understood. We have therefore undertaken the present study to characterize spheroid cells from ascites in comparison with tumor cells from metastatic sites to gain new insight into the mechanisms promoting HGSC cell attachment, including a comprehensive analysis of the currently poorly defined genetic relationship between cancer cells from ascites and metastatic lesions.

## 2 Materials and Methods

### 2.1 Ascites and cells isolated from ovarian cancer patients

Ascites was collected from untreated patients with HGSC prior to surgery at Marburg University Hospital. All patients received the same standard chemotherapy regimen (paclitaxel plus carboplatin) after surgery. Tumor tissue was isolated by microdissection (see below for details) of metastases from matched patients (Table S1). The collection and analysis of human material were approved by the ethics committee at Philipps University (reference number 205/10). Donors provided written consent in accordance with the Declaration of Helsinki. Primary tumor cell cultures (termed OCMI tumor cells) were established from ascites tumor spheroids according to Ince et al. [34] with modifications, as previously reported [35]. This culture system allows for the propagation of ovarian cancer cells over long periods of time in the absence of culture‐induced crisis or new genetic alterations as compared to the original tumor. In the present study, we used OCMI cells from four different patients, referred to as OCMI38, OCMI121, OCMI122, and OCMI137. In some experiments, we also used the verified HGSC cell line OVCAR8 [36] obtained from the NIGMS Human Genetic Cell Repository of the NIH and cultured in RPMI 1640 (Life Technologies, Darmstadt, Germany) complemented with 10% FBS (Capricorn Scientific, Ebsdorfergrund, Germany).

### 2.2 Flow cytometry

Calretinin expression in tumor cells was analyzed by flow cytometry (FACSCanto II, BD Biosciences, Heidelberg, Germany) as described previously [8] using the anti‐calretinin (Santa Cruz Biotechnology, Heidelberg, Germany) and anti‐mouse FITC antibody (eBioscience/Thermo Fisher Scientific, Dreieich, Germany). Results were calculated as percentage of calretinin‐positive cells. Apoptosis was measured using the Annexin V FITC apoptosis detection kit (BD Biosciences) according to the manufacturer’s protocol, but using Annexin V APC antibody (BD Biosciences).

### 2.3 Tumor cell adhesion

Ninety‐six‐well plates were coated in triplicates with 5 µg·cm^−2^ collagen type I (from rat tail, Thermo Fisher, Dreiech, Germany) diluted with 0.02 m acetic acid overnight at 4 °C. Wells were washed with PBS, blocked with 1% heat‐inactivated BSA in PBS for one hour at 37 °C, and washed again. 30 000 tumor cells were added per well and allowed to adhere for 2 h at 37 °C. The wells were washed three times with PBS to remove any nonadherent cells. Adherent cells were fixed with 1% glutaraldehyde and stained with 0.1% Crystal Violet, and the dye crystals were dissolved in 10% acetic acid as described [37]. The optical density, which correlates with the cell number, was measured at 595 nm.

### 2.4 Immunohistochemical staining of HGSC metastases and spheroids

For immunohistochemistry, heat‐induced epitope retrieval was performed either with citrate or with EDTA buffer according to the manufacturer’s protocol of the respective primary antibody. Staining was performed on a Leica BOND‐MAX. Sections were incubated for 1 h with the following primary antibodies: mouse monoclonal anti‐calretinin antibody (Dako M 7245, clone DAK Calret 1; 1 : 100), mouse monoclonal anti‐EPCAM antibody (Ber‐EP4; Dako M 0804, Clone Ber‐EP4; 1 : 50), and mouse monoclonal SMA antibody (Progen Biotechnik GmbH, Clone ASM‐1; 1 : 200). Sections were incubated with the primary antibody for 20 min, except for SMA (30 min). Specimens were washed and incubated with postblock solution and HRP‐polymer reagent according to the manufacturer’s protocol (ChromoPlex 1 Dual Detection Kit for BOND; Leica). For TWIST and p53, staining was performed on a DAKO Autostainer Plus. Sections were incubated for one hour with mouse monoclonal anti‐TWIST antibody (1 : 200; Abcam ab175430, Berlin, Germany, Clone 10E4e6) or mouse monoclonal p53 antibody (1 : 50; Dako M 7001, Clone DO‐7). Sections were washed and incubated with postblock solution and HRP‐polymer reagent according to the manufacturer’s protocol of ZytoChem Plus HRP Polymer Kit (Zytomed Systems, Bargteheide, Germany).

### 2.5 RNA Isolation and qRT‐PCR

RNA isolation, cDNA synthesis, and qRT‐PCR analyses were performed as described previously [38, 39]. *RPL27* was used for normalization. qRT‐PCR was carried out using the following primers: *RPL27*, AAAGCTGTCATCGTGAAGAAC and GCTGTCACTTTGCGGGGGTAG; *CALB2*, CGAGTTTATGGAGGCTTGGC and AAGTTTTCCTGGACAGGCAGG; and *PDPN*, CACTCCACGGAGAAAGTGG and GAGTACCTTCCCGACATTTTTC. Raw data were evaluated with the Cy0 method [40].

### 2.6 siRNA transfection

siRNA transfection was performed using the TransIT‐X2 reagent (Mirus Bio) according to the manufacturer’s protocol. Calretinin knock down was performed using equimolar mixtures of three siRNA oligonucleotides (Sigma‐Aldrich, Taufkirchen, Germany): #1 (5’‐GUCAAAGAGUGACAACUUU‐3’), #2 (5’‐CGCAGAUCCUGCCAACCGA‐3’), and #3 (5’‐CCCUAAUUCUCUUCGCUGU‐3’). MISSION siRNA Universal Negative Control #1 from Sigma‐Aldrich was used as a control. Cells were harvested 96 h after transfection.

### 2.7 OC panel design for targeted sequencing

Genes recurrently altered in OC were selected from published whole‐genome, whole‐exome, and RNA‐Seq studies [41, 42, 43, 44, 45] (Table S2). Probe design was performed with NimbleDesign (Roche, Mannheim, Germany). The panel covers the complete coding regions of 17 genes, 3 complete genes, and from one gene exonic and intronic regions, which are affected by SV, and encompasses 667 kb.

### 2.8 Library generation and sequencing of microdissected samples

Tumor areas were microscopically selected on HE‐stained slides, and the marked region was isolated from serial 5‐µm‐thick unstained sections by microdissection to obtain a tumor cell content (>70%) as assessed microscopically prior to DNA extraction (except for the lymph node metastasis of patient OC122 with an uncertain fraction of tumor cells). The SeqCap EZ HyperCap Workflow (Roche) was used for targeted next‐generation sequencing (NGS). The Kapa High Throughput Library Preparation Kit (Roche) with 1 µg of sonicated gDNA with an average length between 180 and 220 bp (Covaris M220) was used for end repair and A‐tailing, adapter ligation, and library amplification. The HyperCap Target Enrichment Kit (Roche) was used for hybridizing the libraries, washing, and amplification of captured libraries. MiSeq Reagent v3 600‐cycle kit and a MiSeq were used for paired‐end sequencing (2 × 150 cycles).

### 2.9 Analysis of NGS data

For detection of SNVs, indels, and SVs, the Enrichment v3.0.0 (using Isaac aligner) and Variant Studio v3.0 app of the Illumina Base Space Sequence Hub were used. For variant calling, only unique reads as defined by individual start and end points of fragments were used [46]. Variants causing nonsynonymous changes, with sequencing quality score of at least 30 and at least 2 unique variant reads, were used for analysis. The ExAc (Exome Aggregation Consortium) was used to exclude common single nucleotide polymorphism (SNP) with population allele frequencies> 0.1%. All variants were inspected using the integrative genomics viewer software (Broad Institute, Cambridge, MA, USA).

### 2.10 RNA‐Seq data

RNA‐Seq data for spheroids from HGSC ascites used for Fig. 3A and B and Tables S4–S7 have been published previously [17, 38]. Data for OCMI cells have been deposited at EBI ArrayExpress (accession: E‐MTAB‐8902). Transcriptome data for primary pleural and peritoneal mesothelial cells in Fig. 3B have previously been reported [47].

### 2.11 Immunoblotting of p53 and p21

OCMI cell lines were cultured in the presence of 10 µm Nutlin‐3a (Sigma) or DMSO as solvent control. Cells were lysed after 24 hrs of treatment and analyzed by Immunoblotting as described [48] using the following antibodies: anti‐p53 (DO1, gift from B. Vojtesek,1 : 10 000), anti‐p21 (#sc‐6246, Santa Cruz; 1:250), and anti‐β‐actin as loading control (AC‐15, #ab6276, Abcam; 1 : 2500).

### 2.12 Statistical analysis

Comparative data were statistically analyzed by unpaired Student’s t‐test (two‐sided, equal variance). Significance levels are indicated as ****, ***, **, and * for *P* < 0.0001, *P* < 0.001, *P* < 0.01, and *P* < 0.05, respectively. Box plots in Figs 3 and S3 were constructed using the Seaborn boxplot function with Python. Associations with relapse‐free survival (logrank test), hazard ratio (HR), and median survival times were analyzed using the Python Lifelines KaplanMeierFitter and CoxPHFitter functions. All logrank test results are presented as nominal p‐values. The data in Fig. 6 were obtained from the PRECOG [49] and Kaplan–Meier Plotter [50] meta‐analysis databases and The Cancer Genome Atlas (TCGA) [43].

## 3 Results

### 3.1 Comparison of genetic alterations in ascites‐derived tumor cells, ovarian tumor masses, and peritoneal metastases

**Fig. 1:**
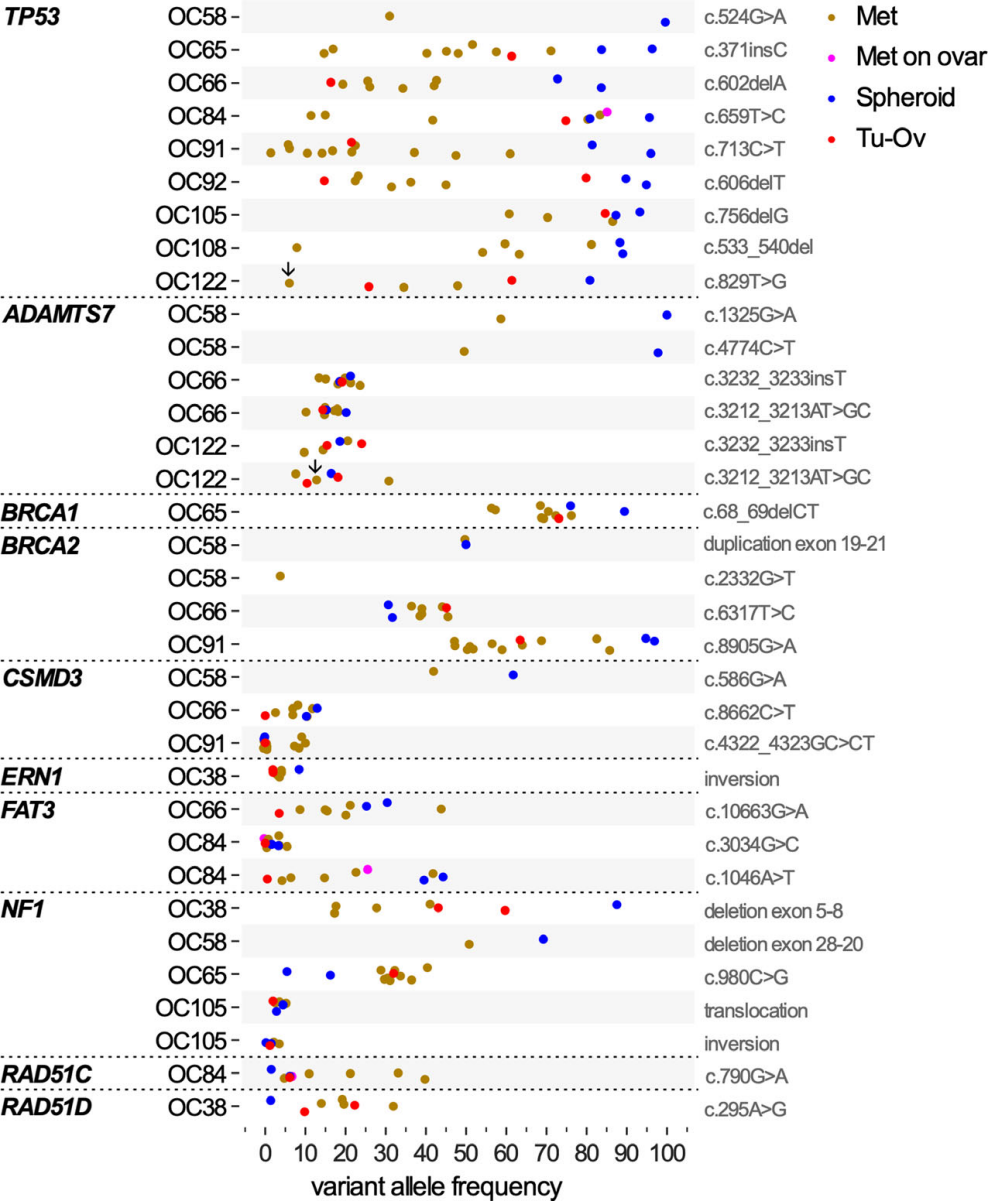
Patterns of genetic alterations in tumor cell spheroids, ovarian lesions, and metastases. HGSC‐associated genetic loci were analyzed in samples from 10 HGSC patients (denoted OC38 to OC122) by targeted sequencing, which revealed alterations in 9 loci (*ADAMTS7, BRCA1, BRCA2, CSMD3, ERN1, FAT3, NF1, RAD51C,* and *TP53*). The figure shows the variant allele frequency (percentage) of single nucleotide variants (SNVs, including indels) and structural variants (SVs, i.e., deletions, inversions, and translocations) in tumor cell spheroids from ascites (Sph; blue), lesions on ovaries or fallopian tubes (T‐Ov; green), and metastasis from different locations (Mets; red). The frequency of *BRCA2* duplication in OC58 was estimated as ~ 50%. All samples contained > 70% tumor cells except for the lymph node from patient OC122 with an uncertain fraction of tumor cells. The corresponding data points for *TP53* and *ADAMTS7* are marked by downward arrows.

In the first part of this study, we sought to investigate whether the seeding of metastases in HGSC patients might be accompanied by the acquisition of genetic alterations in proposed driver genes. Toward this goal, we studied the genetic relationship between detached cancer cells in the peritoneal fluid and cancer cells in tumor tissue by analyzing matched samples of tumor cell spheroids from ascites (*n* = 17), presumed primary tumors (*n* = 10) and metastases (*n* = 52) from 10 HGSC patients (details in Table S1). Using the SeqCap EZ hyperCap workflow for targeted sequencing, we analyzed 21 loci previously associated with single nucleotide variants (SNV), small insertions/deletions (indels), and structural variants (SV) in OC [41] [42, 43] [44, 45] (Table S2). This analysis identified SNVs, including single nucleotide insertions and deletions (indels) in 8 loci, that is, *ADAMTS7, BRCA1, BRCA2, CSMD3, FAT3, NF1, RAD51C,* and *TP53*, in at least one patient (Fig. 1,Table S3). In addition, we found larger deletions, duplications, translocations, and inversions at the *BRCA2, ERN1,* and/or *NF1* loci. While *TP53* mutations occurred in 9 of10 patients, all other SNVs occurred with a much lower prevalence and in different patients (1 to 3 cases out of 10). *TP53* was affected by different structural alterations in different patients, including hot spot mutations (OC58, OC64, OC91, OC122) and indels (OC65, OC66, OC92, OC105, OC108).

**Fig. 2:**
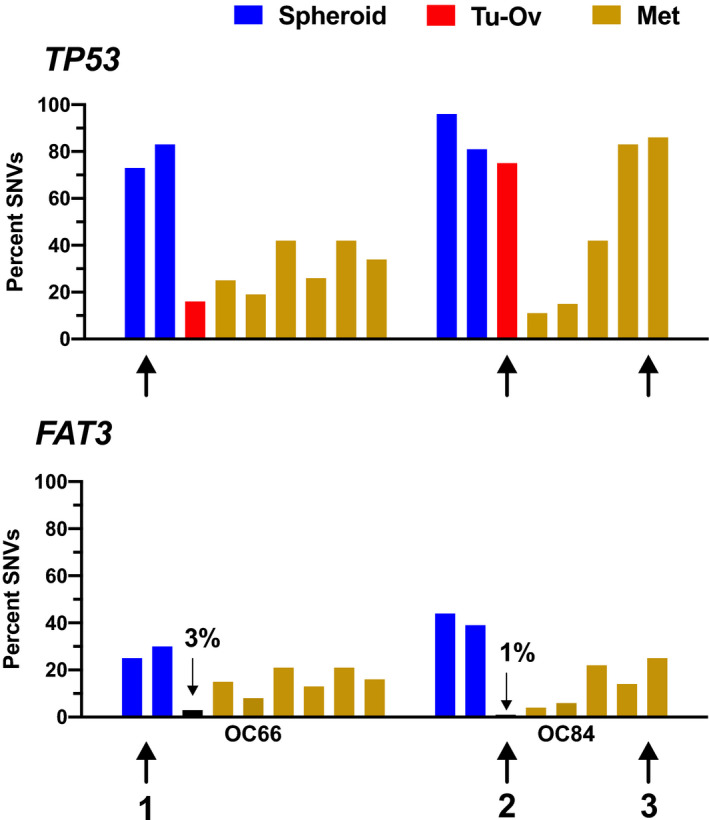
Single nucleotide variations (SNVs) in *TP53* and *FAT3* in tumor cells from different locations in four HGSC patients. Genetic loci were analyzed as in Fig. 1. The bar graph shows the percentage of SNVs in tumor cell spheroids from ascites (Sph; blue), lesions on ovaries or fallopian tubes suspected as primary sites (T‐Ov; red), and metastasis from different locations (Mets; dark yellow). Order of solid tumor samples from left to right is as in Table S1 (columns E/F). For frequencies < 5% the actual percentage is shown. Bars are aligned to allow for a direct comparison of SNV frequencies at different loci in the same sample. Black arrows point examples of strong differences between loci in the same sample. 1: *TP53* versus *FAT3* in spheroids (blue); 2: *TP53* versus *FAT3* in ovarian lesion (red); and 4: *TP53* versus *FAT3* in metastasis (dark yellow).

Importantly, this analysis revealed identical genetic alterations at each locus analyzed in tumor cell spheroids from ascites, ovarian tumor masses, and metastases for all 10 patients (Fig. 1). There were, however, considerable differences in the fraction of cells with specific genetic alterations among samples from the same patients, including differences among solid tumor masses from different locations (Figs 1 and 2). The most extreme genes in this respect were *TP53* (1–96%) and *RAD51D* (1–32%).

It is unlikely that the observed differences in variant allele frequencies merely reflect different proportions of host cells. First, all samples were isolated by microdissection were obtained from tumor regions with a low proportion of stroma and contained > 70% tumor cells (with the exception of the lymph node from patient OC122 with an uncertain fraction of tumor cells). Second, immunostaining for mutant p53 showed a high heterogeneity within the same and among different metastases from the same patient. This is exemplified for patient OC122 with a stabilizing C122G mutation [51]. As shown in Fig. S1, one metastasis showed large areas of either positive or negative p53 staining, presumably originating from subclones differing in their *TP53* status. As the C122G mutation renders p53 nonfunctional [51], it is likely that a new subclone arose by loss of the mutated allele. Third, we repeated the microdissection of tumor samples from the patient with the highest variability of *TP53* mutations alluded to above, which yielded very similar results (not shown), attesting to the precision of the methodology. Fourth, we often found large differences in SNV frequencies for different loci in the same sample (Fig. 2; marked by arrows), for instance, for *TP53* and *FAT3* in spheroids of patient OC66 (73–83% and 25–30%) and for the ovarian lesion (75% and 1%) and metastases 4 and 5 of patient OC84 (83–96% and 14–25%). On the basis of these observations, we conclude that the observed intrapatient differences in SNV frequencies reflect the presence of genetically different tumor cell populations within each lesion rather than highly variable fractions of nontumor cells.

Several of the observed quantitative difference deserve particular attention with respect to a potential role in peritoneal dissemination, in particular *FAT3*, which encodes an untypical cadherin [52, 53]. Cells with mutated *FAT3* are strongly enriched in both spheroids (variant allele frequency 25 and 41%, respectively) and metastases (up to 41%) relative to the presumed primary lesions (1–3%) in two patients (OC66 and OC84; Figs 1 and 2). Likewise, *RAD51C/D* mutations were very low in spheroids (1–6%), and strongly enriched in several metastases (OC38 and OC64; Fig. 1). It is therefore possible that selection of preexisting cells with *FAT3* or *RAD51* mutations played a role in peritoneal dissemination.

### 3.2 OCMI cells genetically resemble the parental HGSC cells

The genetic resemblance of spheroids and metastases validates the use of tumor cells isolated from ascites for studying HGSC biology. We therefore asked whether the same applies to tumor cell cultures established from ascites‐derived cells using a method reported to maintain the genotype of OC cells [34] (subsequently referred to as OCMI cells). To this end, we determined SNVs in OCMI cells from HGSC patients. As shown in Table 1, we found the same SNVs in spheroids from ascites and OCMI cells, that is, in *RAD51D* in OCMI38 (T99A; 14% in spheroids; 49% in OCMI cells), in *ADAMTS7* in OC122 (N1071S and Y1078fs; 17 and 18% in spheroids; 14 and 17% in OCMI cells), and in *TP53* (R248W; 93% in spheroids; 96% in OCMI cells). The *TP53* SNV in spheroids from patient 122 (C277G) was not detected in OCMI122 cells, in contrast to the *ADAMTS7*‐mutated allele. Tumor cells with this genotype also appear to be present in metastatic lesions from the same patient, as suggested by the high variation in *TP53* variant allele frequency (6–48%), but very similar ADAMTS7 frequency (14–18%; Fig. 1; Table 1). A similar situation seems to exist for patient OC38, where an *NF1* deletion was found in metastases with a frequency of 10–32%, but was not detectable in OCMI38 cells (not shown), whereas the same *RAD51D* mutation was detected in all samples with the highest frequency in cultured cells (10–32% in metastases and spheroids; 49% in OCMI38 cells).

**Table 1:** Single nucleotide variants (SNVs) in solid tumor lesions (ovarian masses and metastases), tumor cell spheroids from ascites, and cultured OCMI cells from the same spheroids. Samples from 3 patients were included (OC38, OC122, and OC137). Solid tumor lesions were not analyzed for OC137.

Sample	Locus	Amino acid altered	SNV frequency
*OC38*			
Solid tumor lesions	*RAD51D*	T99A	0.10 – 0.32
Spheroids from ascites	*RAD51D*	T99A	0.014
Cultured cells (OCMI38)	*RAD51D*	T99A	0.49
*OC122*			
Solid tumor lesions	*ADAMTS7*	N1071S	0.08 – 0.20
	*ADAMTS7*	Y1078fs	0.10 – 0.24
	*TP53*	C277G	0.06 – 0.48
Spheroids from ascites	*ADAMTS7*	N1071S	0.17
	*ADAMTS7*	Y1078fs	0.18
	*TP53*	C277G	0.80
Cultured cells (OCMI 122)	*ADAMTS7*	N1071S	0.14
	*ADAMTS7*	Y1078fs	0.17
	*TP53*	C277G	0.00
*OC137*			
Spheroids from ascites	*TP53*	R248W	0.93
Cultured cells (OCMI 137)	*TP53*	Mutated (p53 protein stabilized; see Fig. S2)	

The C277G SNV in *TP53* in OCMI122 cells is a loss‐of‐function mutation [51], which might explain its low frequency in some metastases and its loss in the OCMI122 cell population. In contrast, the R248W SNV found in OC137 spheroids (Table 1) is a known hot spot driver mutation in HGSC [54], and accordingly, mutated (stabilized) p53 protein was found in OCMI137 cells as opposed to OCMI38 cells with wild‐type *TP53* (Fig. S2). Based on these findings, it can be ruled out that OCMI cells were derived from cancer‐associated fibroblasts, since the latter are not affected by genetic alterations [55]. Furthermore, irrespective of the *TP53* status, all three OCMI cell populations showed strong invasive properties comparable to established HGSC lines (e.g., OVAR8; data not shown) in an *in vitro* 3D matrigel model [35] using ascites as chemoattractant. Taken together, these findings suggest that OCMI cells established from HGSC spheroids represent a suitable experimental model for studying metastasis‐associated processes and were therefore used in subsequent experiments.

### 3.3 Induction of both mesothelial and mesenchymal marker genes in adherent HGSC cells

**Fig. 3:**
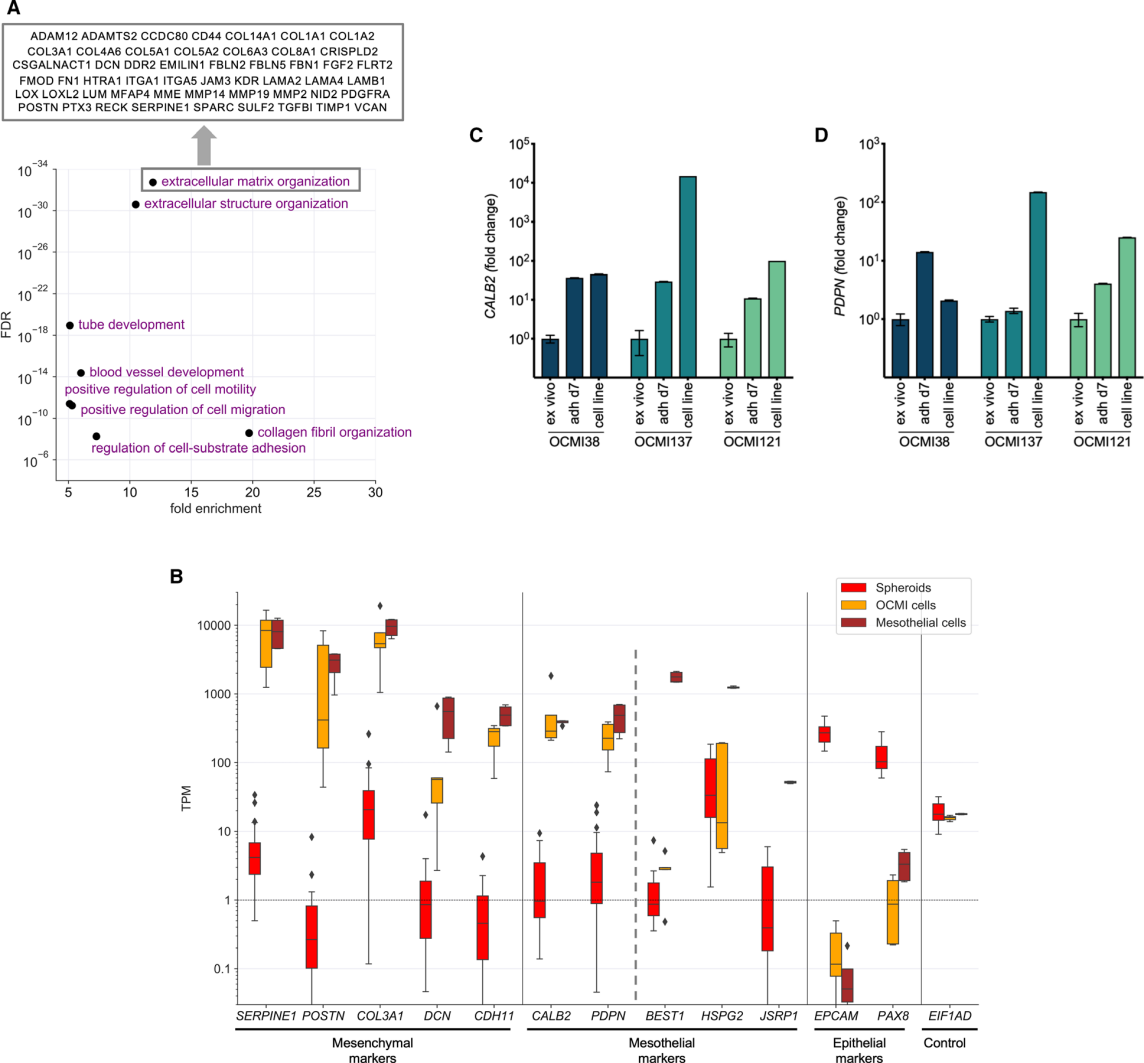
Comparative analysis of the transcriptomes of tumor cell spheroids from ascites, adherent spheroid‐derived cells in culture, and tumor tissue from HGSC patients. (A) Gene ontology (GO) term enrichment analysis of genes upregulated in adherent spheroid‐derived cells versus tumor cell spheroids *ex vivo* (Table S4), indicating enrichment for ECM remodeling, cell migration, and adhesion (list of ECM‐associated genes in Table S6. (B) Examples of mesenchymal, mesothelial, and epithelial marker genes up‐ or downregulated in adherent spheroid‐derived cells. The plot includes RNA‐Seq data for primary pleural and peritoneal mesothelial cells (*n* = 4) [47] for comparison. Box plots show median (line), upper and lower quartiles (box), range (whiskers), and outliers (diamonds). (C) qRT‐PCT analysis of *CALB2* and *PDPN* expression in tumor cell spheroids from ascites (*ex vivo*), 7 days after plating spheroids in culture (attached cells) and in cells from these cultures after multiple passages (cell line). Data were normalized to *RPL27* Cy0 values and set to 1 for *ex vivo* tumor cell spheroids. Data are shown for 3 patients with 3 triplicates each (mean ± standard deviation).

To shed light on the molecular events accompanying adhesion of HGSC cells during peritoneal dissemination, we determined the transcriptomes of adherent OCMI cells and tumor cell spheroids. This analysis identified 268 genes significantly upregulated in adherent cells with a minimal median fold change (FC) of 10 (Table S4), and 459 significantly downregulated genes (FC ≥ 10; Table S5). The group of upregulated genes encompasses a large number of mesenchymal markers, such as collagens, laminins, matrix metalloproteinases (*MMPs*), serine peptidase inhibitors (*SERPINs*), other matrix proteins, and transcription factors, such as *ZEB1* and *ZEB2* (Table S4). Consistent with these observations, gene ontology (GO) term enrichment analysis of the upregulated genes showed that ECM remodeling, cell migration, and adhesion were the most significant specific biological functions (Fig. 3A). Table S6 lists the upregulated ECM remodeling‐associated genes identified by the GO term analysis.

In contrast, the group of downregulated genes contained numerous genes associated with cell motility (e.g., genes coding for Rho GTPase‐activating proteins of the *ARHGAP* family), cell cycle‐regulated genes, and epithelial markers, such as *EPCAM, ERBB3, MUC16,* and *PAX8* (Table S5). In agreement with the downregulation of *ARHGAP* genes, GO term enrichment analysis of the downregulated identified ‘regulation of GTPase activity’ as the strongest hit (*P* < 10^−5^). Intriguingly, *BRCA1* and *BRCA2* were also found in the group of downregulated genes, which may be relevant with respect to therapy.

Examples of mesenchymal and epithelial marker genes up‐ or downregulated in adherent spheroid‐derived cells are shown in Fig. 3B. Surprisingly, the upregulated genes also included several mesothelial markers, such as calretinin (*CALB2*) and podoplanin (*PDPN*; Table S4), which reach expression levels close to primary mesothelial cells (Fig. 3B). This was confirmed by comparative qRT‐PCR analysis of patient‐derived tumor cell spheroids (directly after isolation from ascites) and attached cells outgrown from these spheroids (7 days after plating in culture), which showed a strong upregulation of *CALB2* and *PDPN* in the attached cells from all three samples analyzed (up to 37‐fold; Fig. 3C). Upon further passaging, *CALB2* and *PDPN* expression increased further in two of these cases. In contrast to *CALB2* and *PDPN*, a large number of genes expressed highly in mesothelial cells (TPM > 400) were expressed at much lower levels in adherent OCMI cells (*n* = 113 for FC ≥ 5; Table S7; *BEST1, JSRP1,* and *LDLRAD2* as examples in Fig. 3B), indicating that attachment triggers the upregulation of a small, specific subset of mesothelial genes rather than the conversion to a mesothelial transcriptional phenotype.

### 3.4 Upregulation of the CALB2 gene and calretinin protein expression in HGSC metastases

We next addressed the potential *in vivo* relevance of the data reported above. To this end, we asked whether mesenchymal and mesothelial marker genes are also upregulated in solid tumor tissue relative to tumor cell spheroids from ascites, making use of published RNA‐Seq datasets [41]. As shown in Fig. S3, expression of the top 15 genes upregulated in attached OCMI cells (identified above) was also increased in tumor tissue relative to spheroids, including *CALB2* and *PDPN*. It is unlikely that the higher expression of mesothelial and mesenchymal genes in tumor tissue originates from host cells, since the immune cell markers *CD4, FCGR3A (CD16), CD14,* and *CD163* and the mesothelial markers *BEST1* and *HSPG2* are expressed at similar levels in spheroids and tumor tissue (Fig. S3).

**Fig. 4:**
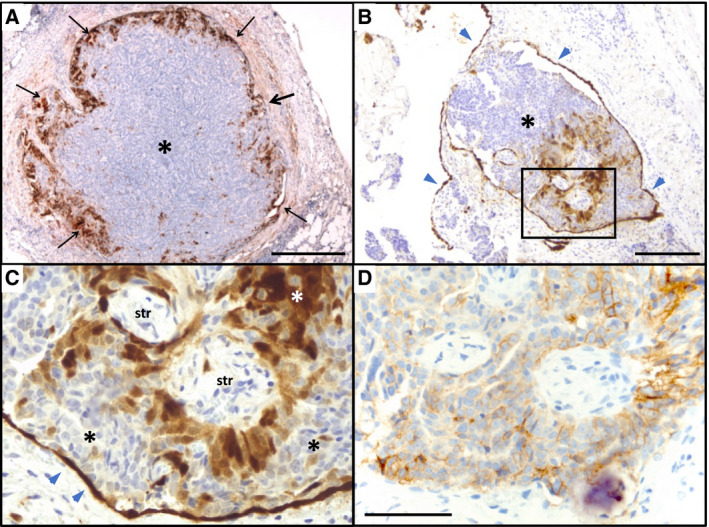
Immunohistochemical staining of peritoneal HGSC metastases. (A, B) Examples of peritoneal metastases showing calretinin expression, particularly on the invasive front (black arrows in panel A). (C–E) Higher magnification of the section marked in panel B stained for calretinin (C) or the tumor cell marker EPCAM (D). Blue arrowheads in B and C indicate the intact peritoneal mesothelium, which is strongly positive for calretinin and negative for EPCAM. *: tumor cells; str: stroma cells. Scale bars: 500 µm (A), 200 µm (B), and 50 µm (C, D).

In agreement with this notion, immunohistochemical staining of metastases from HGSC patients showed strong calretinin staining in subsets of tumor cells (Fig. 4A,B). Calretinin‐positive cells were identified as tumor cells based on their morphology and expression of the carcinoma cell marker EPCAM (Fig. 4D). Calretinin‐expressing tumor cells frequently accumulated at the tumor edges (Fig. 4A), but occurred also inside the tumor in particular in areas of interspersed stroma (Fig. 4C). A summary of the analysis of 124 metastases from 20 patients is shown in Table 2.

**Table 2:** Results of immunohistochemical calretinin staining of HGSC metastases and spheroids form ascites. Calretinin‐negative samples are highlighted (shaded fields). CR, calretinin; n.a., not applicable; n.d., not done.

Pat. ID	Site	CR‐pos. (*n*)/all mets. (*n*)	Total n of cells counted in CR‐pos. metastasis	CR‐pos. cells in metastasis [*n* (%)]	Location of CR‐positive cells	CR‐pos. spheroids/total n of spheroids (% pos.)	CR‐positive cells in CR‐pos. spheroids (total *n*/positive n)
26	Pelvis minor	1/2	n.d. (>4mm)	n.a.	n.a.	26/1338 (1.94%)	50/314 (15.9%)
	Pelvis minor	0/12	n.a.	n.a.	n.a.		
	Colon	1/2	4713	11 (0.23%)	tumor edges		
27	Omentum majus	1/2	n.d. (>4mm)	n.a.	tumor edges^a^	0	0
	Peritoneum	5/11	6655 1375 5720 2x n.d. (>4mm)	45 (0.68%) 10 (0.73%) 4 (0.07%) n.a.	heterogenous tumor edges heterogenous heterogenous		
37	Rektum	2/3	2435 n.d. (>4mm)	8 (0.33%) n.a.	heterogenous	21/147 (14.3%)	28/1701 (1.64%)
	Colon	4/5	2434 3x n.d. (>4mm)	5 (0.21%) n.a.	central heterogenous		
44	Adnex	0/1	2434	n.a.	n.a.	15/1055 (1.42%)	20/146 (13.7%)
	Colon	1/7	615	6 (0.98%)	heterogenous		
54	Colon	0/7	n.a.	n.a.	n.a.	19/838 (2.27%)	36/431 (8.35%)
	Colon	1/3	n.d. (>4mm)	n.a.	heterogenous		
66	Pelvic wall	0/10	n.a.	n.a.	n.a.	7/245 (2.86%) and 10/>86 (11.6%)	14/326 (4.29%
67	Abdominal wall	1/13	1462	34 (2.33%)	heterogenous	16/636 (2.52%)	17/103 (5.67%)
84	Externa	3/6	1954 1180 1086	15 (0.77%) 6 (0.51%) 13 (1.20%)	tumor edges heterogenous heterogenous	2/42 (4.76%)	2/28 (7.14%)
91	Paracolic gutter	1/8	410	12 (2.93%)	tumor edges^a^	n.d.	n.a.
92	Paracolic gutter	2/5	2166 2240	58 (2.68%) 18 (0.80%)	tumor edges^a^ heterogenous	n.d.	n.a.
105	Colon	5/6	6259 9137 3x n.d. (>4mm)	255 (4.07%) 51 (0.56%)	heterogenous tumor edges^a^ heterogenous	8/530 (1.51%) and 5/21 (23.8%)	20/99 (20.2%)
108	Paracolic gutter	1/8	351	33 (9.40%)	tumor edges	3/125 (2.40%) and 0	5/112 (4.46%)
114	Abdominal wall	4/7	449 851 15 355	170 (37.9%) 39 (4.58%) 3 (20.0%) 4 (1.13%)	tumor edges tumor edges tumor edges tumor edges	39/628 (6.21%) and 38/278 (13.7%)	528/5095 (10.4%)
122	Paracolic gutter	0/5	n.a.	n.a.	n.a.	8/374 (2.14%)	7/96 (7.29%)

^a^ Tendency toward location at tumor edges.

**Fig. 5:**
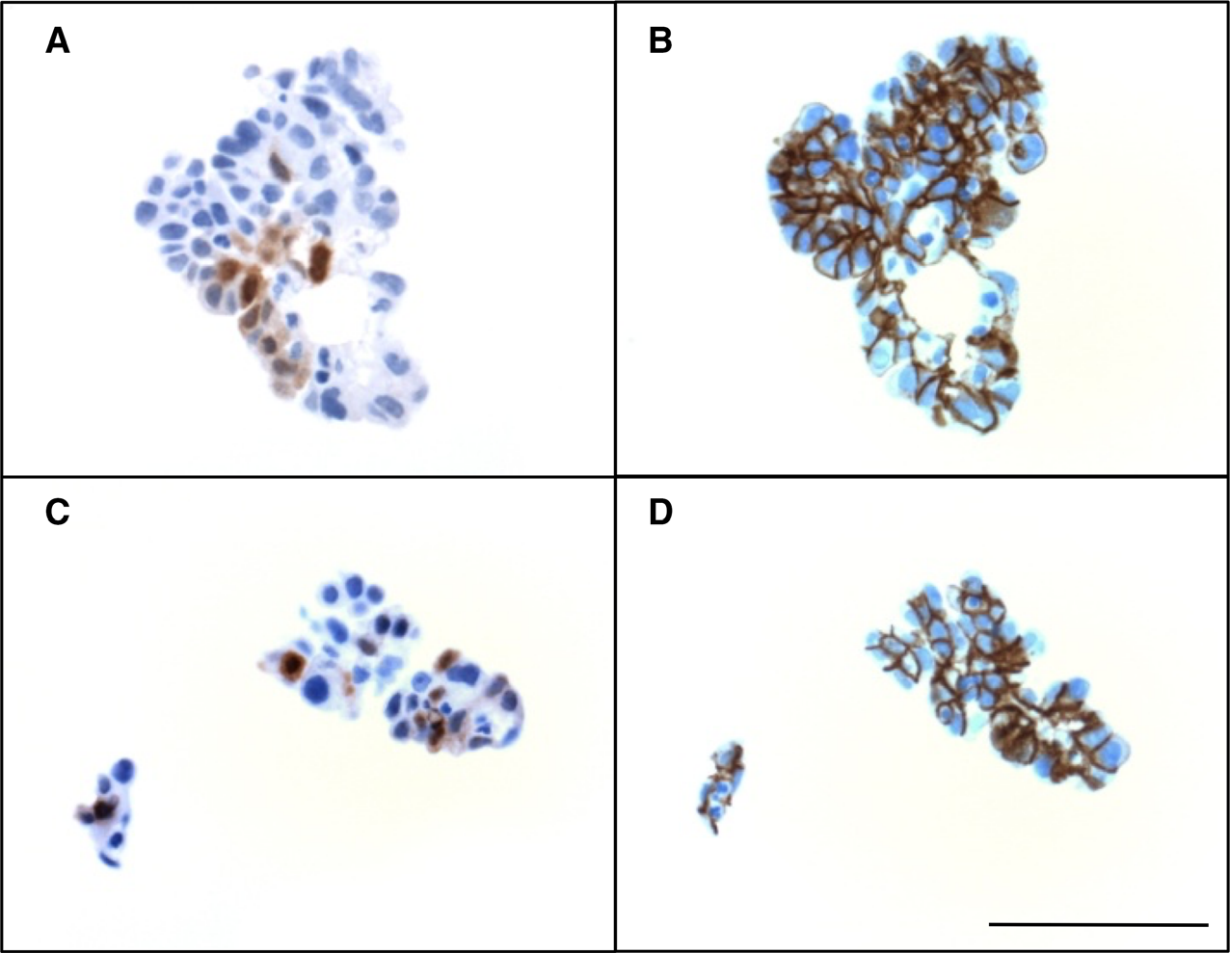
Immunohistochemical staining of rare tumor cell spheroids with calretinin‐expressing cells in ascites from 2 different HGSC patients. (A, C) Spheroids showing focal expression of calretinin. (B, D) EPCAM staining of the same spheroids showing that calretinin‐expressing cells are carcinoma cells. Scale bar: 50 µm.

Calretinin staining was also found in spheroids from ascites from all but one patient (Table 2). The percentage of calretinin‐positive spheroids was highly variable among patients ranging from 1.9 to 23.8% (Table 2; penultimate column). Likewise, the percentage of calretinin‐positive cells in calretinin‐positive spheroids varied over a range of ~ 1–80% (Table 2; last column). Figure 5A and C shows two examples of spheroids with ~ 20% calretinin‐positive cells. Both morphological criteria and positivity for EPCAM (Fig. 5B,D) strongly suggest that these cells are tumor cells.

As shown in Fig. S4, tumor cells in metastases also strongly expressed the mesenchymal markers smooth muscle actin (SMA/ACTA2) and nuclear TWIST, while EPCAM expression was weaker compared with tumor cells in spheroids (Fig. 5B,D). SMA was not detectable in spheroids (not shown), whereas TWIST was clearly expressed in a subset of tumor cells (Fig. S5). These findings are consistent with our *in vitro* findings (Section 3.3) and support the hypothesis that metastasizing HGSC cells undergo transition to a mesenchymal–mesothelial phenotype. However, there appears to be considerable heterogeneity with respect to coexpression of mesenchymal and mesothelial makers, with calretinin showing the most pronounced restriction.

### 3.5 Association of high CALB2 and PDPN expression levels with a poor clinical outcome of HGSC

**Fig. 6:**
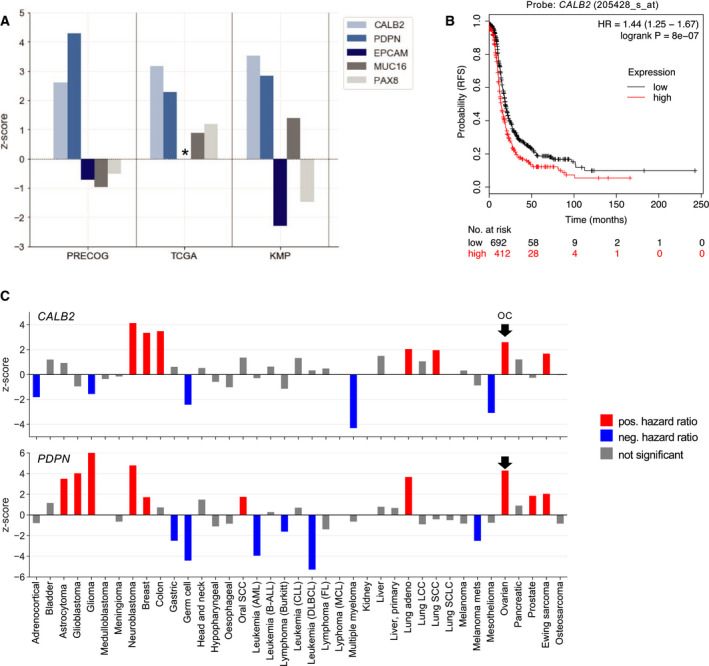
Association of *CALB2* and *PDPN* with short relapse‐free (RFS) and overall (OS) HGSC survival. (A) z‐scores for *CALB2* and *PDPN* and for comparison 3 epithelial markers based on data from the PRECOG and KM Plotter meta‐analysis databases [49, 50] and the TCGA microarray dataset [43] (PRECOG: OS of OC,TCGA: RFS of OC; KMP: RFS of HGSC. z‐score above 1 indicates a hazard ratio (HR > 1 (poor outcome; *z*‐score below −1 indicates a HR < 1 (favorable outcome. A z‐score of 1.96 or −1.96 corresponds to a logrank p value of 0.05. (B The Kaplan–Meier plot showing the association of CALB2 expression and a poor RFS (generated by Kaplan–Meier Plotter, 2017 version; http://kmplot.com). (C) *z*‐scores for *CALB2* and *PDPN* across all cancer entities in the PRECOG database. Red: *z*‐score above 1.96 indicating a poor clinical outcome; blue: *z*‐score below −1.96 indicating a favorable outcome; gray: *z*‐score < 1.96 and >−1.96 (i.e., *P* < 0.05). The arrows point to OC.

We next addressed the potential clinical relevance or *CALB2* and *PDPN* expression in HGSC cells. To this end, we evaluated the data from the PRECOG and KM Plotter meta‐analyses [49, 50] and The Cancer Genome Atlas (TCGA) dataset [43], which consistently revealed a strong association of high *CALB2* or *PDPN* expression with a short relapse‐free survival (RFS) and overall survival of HGSC, while no significant link of survival to the epithelial markers *EPCAM, MUC16,* and *PAX8* (included for comparison) was observed (Fig. 6A). The Kaplan–Meier plot in Fig. 6B illustrates this in more detail for *CALB2*. We also analyzed the association of *CALB2* and *PDPN* expression with the overall survival (OS) for all other entities in the PRECOG database, which yielded clearly opposing correlations (Fig. 6C). While high *CALB2* or *PDPN* expression in several other carcinomas, including breast cancer, was associated with a poor prognosis, the OS of other cancers, in particular hematologic malignancies, showed the opposite association. This suggests that calretinin and podoplanin have different functions in different cancer entities, which may either be tumor‐suppressive of tumor‐promoting function.

### 3.6 Blockade of HGSC cell adhesion by interference with calretinin expression

The results described above suggest a function for calretinin in spheroid attachment and/or spreading. We therefore interrogated the role of calretinin in tumor cell adhesion to a collagen type I matrix by a siRNA‐based loss‐of‐function approach. As shown in Fig. S6, the 3 selected siRNAs directed at *CALB2* showed a strong inhibition of RNA expression with a > 90% reduction, tested either as single siRNAs or as pooled. Since we could not obtain an anti‐calretinin antibody suitable for immunoblotting, we assessed protein expression by flow cytometry, which showed a clear reduction of calretinin‐positive cells in siRNA‐transfected cells (Fig. S7).

**Fig. 7:**
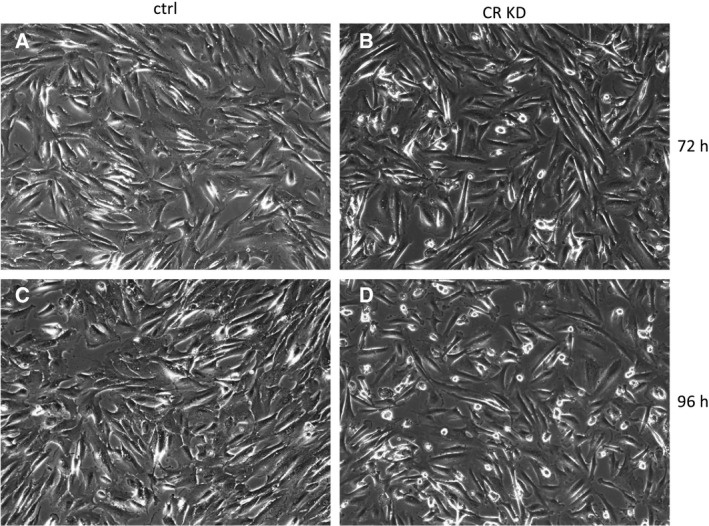
Loss of HGSC cell adherence by interference with CALB2 expression. OCMI137 cells were treated with control (left; ctrl) and *CALB2* (right; CR KD) siRNA pool and observed 72 h (top) and 96 h after transfection under a phase‐contrast microscope. Cells start to detach after 72 h under *CALB2* siRNA treatment, which is strongly increased at 96 h.

**Fig. 8:**
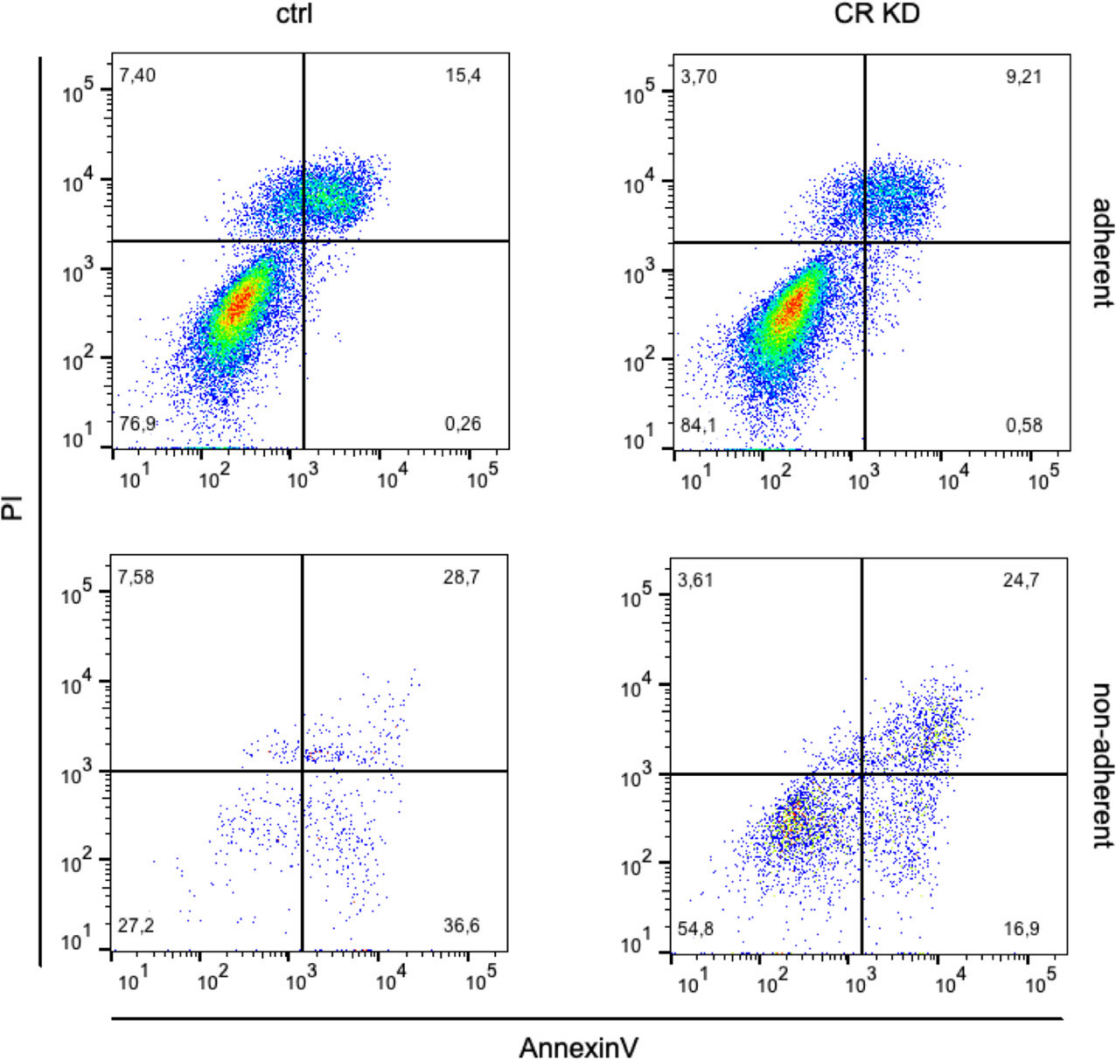
Identification of apoptotic cells upon interference with CALB2 expression in cultured HGSC cells. OCMI137 cells were treated with control (ctrl) or *CALB2* siRNA pool (CR KD) for 96 h, and adherent (top) and detached (bottom) cells were analyzed for cell death by flow cytometry after staining with Annexin V and propidium iodide (PI). Numbers indicate the percentage of cells in each quadrant.

Treatment of OCMI cells with CALB2‐siRNA resulted in cell detachment, first evident 72 h after siRNA transfection and clearly visible after 96 h by increased numbers of cells displaying a rounded morphology or floating in the culture medium (Fig. 7). Flow cytometry analysis of the adherent cell population (including rounded cells) did not show Annexin V or propidium iodide (PI) staining at 96 h after CALB2‐siRNA transfection relative to control siRNA (Fig. 8), indicating that the loss of calretinin expression did not induce cell death in these cells. Analysis of the floating cells clearly identified cells at early stages of apoptosis (Annexin V high, PI low; ~17%) and late stages (Annexin V high, PI high; ~25%), which was similar (or even lower) compared with control siRNA‐transfected cells. Importantly, >50% of floating cells remained unaffected after CALB2‐siRNA transfection. These results clearly suggest that detachment precedes cell death.

**Fig. 9:**
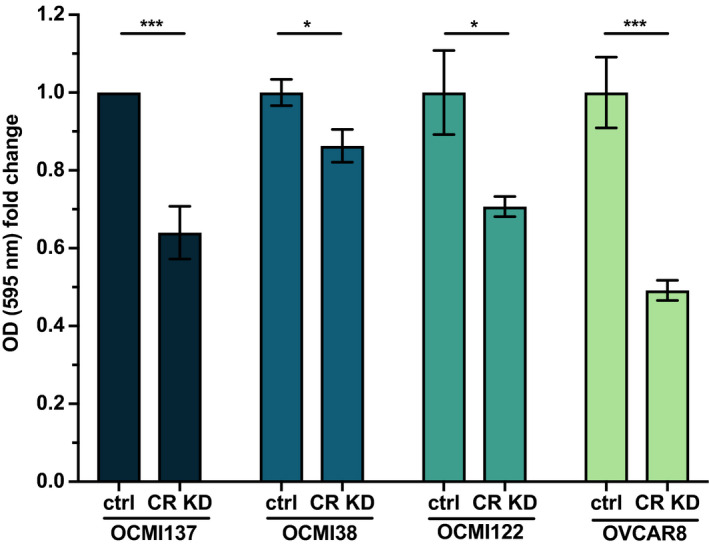
Reduced adherence of HGSC cells to collagen I by interference with CALB2 expression. OCMI cells from 3 different patients and OVCAR8 cells were treated with control (ctrl) or *CALB2* siRNA pool (CR KD) for 96 h, detached from the culture dish and seeded on collagen type I‐coated wells. After 2 h, the adhered cells were stained with Crystal Violet and the optical density was measured at 595 nm. Significance was determined by unpaired, two‐tailed *t*‐test *: *P* < 0.05, **: *P* < 0.01, ***: *P* < 0.001, and ****: *P* < 0.0001. Data are shown for three triplicates each (mean ± standard deviation).

To obtain further evidence for this conclusion, we analyzed the ability of cells to reattach to a collagen type I matrix. To this end, OCMI cells were transfected with control siRNA or CALB2‐siRNA and seeded out on collagen type I‐coated wells 96 h post‐transfection. Cell attachment was assessed by Crystal Violet staining 2 h after seeding and showed a clear reduction for all 3 OCMI cell lines tested (Fig. 9), confirming a pivotal role for calretinin in HGSC cell adhesion. To exclude that this finding reflects a feature of the experimental system (OCMI cells), we performed the same experiment with the established and verified HGSC cell line OVCAR8, which fully confirmed the data obtained with OCMI cells (Fig. 9).

## 4 Discussion

### 4.1 Genetic alterations in HGSC metastasis and spheroids

As it is unknown whether the ability of detached HGSC cells to colonize serous membranes might be linked to newly acquired genetic alterations, we performed targeted sequencing of 21 loci proposed as HGSC driver mutations in 50 samples from 10 patients, consisting of spheroids, ovarian tumor masses suspected as primary lesions, and metastases from different peritoneal locations. As expected, *TP53* mutations were found in 9 of 10 patients [56]. Likewise, *ADAMTS8* was altered by SNVs in the majority of patients (6/10; in 3 cases simultaneously at 2 different sites), while 8 other loci were affected in a smaller subset of patients (1–5 out of 10). Genetic alterations were not detected in 11 out of the 21 loci analyzed.

The most important finding of this analysis is the identification of identical genetic alterations at each locus analyzed in all solid tumor samples (including the presumed primary lesions) and spheroids from the same patient in all 10 cases (Fig. 1). This suggests that metastatic spread does not require additional alterations in any of the potential 21 driver loci analyzed, consistent with the notion that HGSC cells acquire metastatic properties at a very early stage [5]. This is in agreement with previous studies analyzing clonal evolution of OC metastasis, which also showed little qualitative variation in mutated loci among different lesions [57, 58, 59].

While the same genetic alterations were detectable at each of the loci analyzed in all samples from the same patient, the fractions of cell with specific genetic alterations were clearly different for two loci. These include *FAT3* and *RAD51A/C*, which both showed much higher frequencies of mutated alleles in metastases. The *FAT* family consists of 4 genes (FAT1‐4), which have been linked to different cancers [52, 53]. The best studied family members are FAT1 and FAT4, which trigger actin polymerization to promote cell migration, inhibit cell proliferation via activation of Hippo signaling, and/or maintain planar cell polarity [53, 60]. Both genes are downregulated or mutated in different cancers, including glioblastoma, melanoma, acute lymphoblastic leukemia, and breast, head, and neck, pancreatic, colorectal, gastric, ovarian, and hepatocellular carcinomas [52, 53]. In Drosophila, the closely related *Fat* gene has been identified as a tumor suppressor gene [52, 61]. Consistent with these findings, the human *FAT1* and *FAT4* genes have tumor‐suppressive functions, although this may be context‐dependent for *FAT1* [52, 53]. Much less is known about FAT3, but its close structural similarity to other FAT family members and its recurrent mutation in HGSC point a role as a tumor suppressor in HGSC. It is currently not known whether this putative tumor‐suppressive role is related to a function in actin regulation and cancer cell adhesion, in cell proliferation, or in another as yet unidentified process. Other genes enriched in several metastases are *RAD51A* and *RAD51C*. Although the only well‐known function of RAD51 proteins is linked to DNA repair, a metastasis‐promoting function has also been proposed for triple‐negative breast cancer [62], but the underlying mechanism remains enigmatic. A clarification of these open questions has to await the results of future studies addressing the molecular functions of *FAT3* and *RAD51A/C* relevant to HGSC metastasis in detail.

An unresolved issue concerns the differences in variant allele frequencies, which in some cases were unexpectedly high among solid tumor masses from different locations and spheroids from the same patient. A characteristic feature of HGSC is the unusually large number of genes than can be altered by driver mutations, but only a small subset of these mutations are found simultaneously. This suggests that tumorigenesis can be supported by variable subsets of partially exchangeable genes, resulting in a high genetic diversity not only among different patients, but also among different tumor sites in the same patient with the consequence that SNV frequencies within mixed tumor cell populations can vary strongly between different tumor sites. We observed genetic heterogeneity also in spheroids for several genes, as indicated by low SNV frequencies, for instance, in *ADAMTS7, CSMD3, ERN1, FAT3,* and *RAD51C*/D (Fig. 1), supporting the view that tumor lesions are composed of mixed tumor cell populations with varying frequencies of SNVs.

However, such a scenario cannot fully explain the situation observed for *TP53*. On the one hand, genetic alterations of *TP53* are considered one of the earliest events driving HGSC tumorigenesis [56], which is consistent with our observation of *TP53* SNVs in 90% of the patients, including both hot spot and inactivating mutations and indels. On the other hand, the *TP53* variant allele frequency is generally high in spheroids (>80%) but low in some metastases (<10% in three patients). One possible explanation for this observation could be the loss of variant alleles with nonessential functions in metastasis formation, such as the inactivating C277G *TP53* variant in patient OC122, which appears to be absent from a large fraction of tumor cells even within the same metastasis (Fig. S1), possibly due to loss of the mutated nonfunctional allele. Consistent with this finding, we have isolated cancer cells from OC122 spheroids that lack the *TP53* variant but harbor the *ADAMTS7* SNV found in solid tumor lesions (Table 1). Whether a similar scenario applies to other TP53 variants remains an open question that is beyond the scope of the present manuscript and will therefore be the subject of subsequent studies.

### 4.2 A mesenchymal phenotype with mesothelial components in adherent HGSC cells and metastases

Comparative profiling of spheroids from ascites and outgrowing adherent cancer cells revealed a transcriptional switch that resembled mesenchymal–epithelial transition, but unexpectedly also included induction of the mesothelial markers calretinin *(CALB2)* and podoplanin *(PDPN)*. Several lines of evidence suggest that the increase in calretinin and podoplanin expression is relevant to peritoneal dissemination: (a) A similar pattern of upregulated mesenchymal and mesothelial genes was identified in metastatic lesions compared with spheroids from ascites; (b) immunostaining revealed calretinin staining in a subset of cancer cells in HGSC metastases with preference for the invasive tumor front; (c) a high expression of *CALB2* or *PDPN* is strongly associated with a short RFS and OS; and (d) *CALB2* silencing triggered the detachment of adherent HGSC cells *in vitro* and inhibited the adhesion of detached HGSC cells to collagen type I. In agreement with these findings, it has been reported that serum calretinin is a prognostic marker for OC [63].

While the transcriptional phenotype of adherent HGSC cancer cells also showed a clear downregulation of epithelial marker genes, as in classical EMT, this effect was considerably weaker in metastases compared with spheroids. Likewise, the upregulation of mesenchymal genes and *CALB2* and *PDPN* was less pronounced in metastases. These findings could be reconciled by a model, where a phenotype with high mesenchymal, high calretinin/podoplanin, and low epithelial markers occurs only transiently around the time of adherence and in invasive regions of metastases. Established metastases would thus represent a mixture of cancer cells at different stages of peritoneal colonization. This would be consistent with the previously published evidence that cancer cells in metastases undergo mesenchymal–epithelial transition (MET) in different entities [64], including OC [65]. Based on these observations, we put forward the hypothesis that the acquisition of mesothelial functions by HGSC cells contributes to their adhesion to the exposed mesothelial‐basal membrane and therefore facilitate the formation of metastatic lesions.

An unresolved issue is the question at what stage HGSC cells upregulate mesothelial genes. Based on the available data for calretinin, two models could be envisaged. In the first scenario, calretinin is upregulated after attachment of tumor cells to peritoneal organs. In this case, the presence of calretinin‐positive cells in spheroids would not be required for metastatic seeding. This would also be compatible with the lack of calretinin‐positive spheroids in the ascites from one patient, although we cannot rule out the possibility that the number of positive spheroids was below the detection limit. In an alternative scenario, calretinin‐positive cells preexisting in spheroids are instrumental in metastasis formation, and calretinin expression may not be upregulated during adhesion or subsequent steps. This model would be consistent with our observation that *CALB2* expression is OCMI cells is not dependent on adhesion, that is, remains high in detached cells (data not shown). We therefore favor the second model at present, but further experimental evidence is needed to test this hypothesis.

### 4.3 Potential function of calretinin in HGSC metastasis

Calretinin is one of the best‐established markers for the identification of mesothelial cells, but its expression has also been described in testicular Leydig cells, brain neurons, and ovarian theca interna, hilus, and surface epithelial cells [66, 67]. However, the significance of calretinin expression observed in specific areas of the ovary is unclear. Calretinin is used for the histopathological distinction of tumor and mesothelial cells [68, 69] and for the diagnosis of malignant mesothelioma [70]. In combination with EPCAM, it is used for differentiating epithelioid peritoneal mesothelioma from serous papillary carcinoma of the ovary [68]. Its expression has also been described in several other human cancer entities, including breast and ovarian carcinoma [67, 71, 72, 73

]. However, the reported frequency of calretinin‐positive cases in HGSC is low (8%) and of unknown significance [71]. In mesothelioma cells, calretinin is essential for growth and/or survival [74], as well as invasion [75]. Calretinin is a Ca^++^‐binding protein that activates the focal adhesion kinase (FAK) [75]. Its experimental downregulation reduced viability, proliferation, and invasive properties of mesothelioma cells concomitantly with an attenuation of FAK signaling [76]. It is therefore likely that calretinin and FAK interact to promote mesothelioma growth and invasion. Prior to the present study, a potential role for calretinin in OC has not been addressed. Our data identify two novel features of calretinin. First, calretinin expression is upregulated in adhering HGSC cells, and second, calretinin has an essential function in HGSC cell adhesion, which is a prerequisite for transcoelomic metastasis formation. We did not observe direct effects of *CALB2* silencing on cell viability, although a fraction of OCMI cells died after detaching from the culture dish. It remains to be investigated whether the reported prosurvival effect of calretinin in mesothelioma cells is also related to a loss of cell adhesion or an independent feature mediated by the AKT pathway [74].

### 4.4 Potential function of podoplanin in HGSC metastasis

Podoplanin is a mucin‐like transmembrane glycoprotein with multiple functions in development, in the regulation, and/or in the differentiation of mammary stem cells, immune cells, and platelets [77]. High expression of podoplanin has been associated with different autoimmune diseases, inflammation, and cancer cell invasion and metastasis [77, 78, 79, 80]. Podoplanin interacts with its ligand CLEC2, other membrane proteins, including Galectin‐8, heat‐shock protein A9 and CD44, and impinges on signaling pathways that regulate cell proliferation, ECM remodeling, migration, and EMT [77]. It also interacts with intracellular proteins of the ezrin/radixin/moesin family [81] to modulate small Rho GTPases, control contraction of the actomyosin, and promote EMT [77, 78, 82]. Furthermore, podoplanin is shed from lymphatic endothelial cells and forms a complex with the chemokine CCL21 on fibroblast‐like cells, which plays a role in the development of specialized T cells in the thymus [83]. On cancer‐associated fibroblasts, it contributes to perturbing immune surveillance toward cancer cells [84]. In OC, podoplanin has been reported to be highly expressed in tumor cells at the invasive front, which correlated with the extent of lymphatic endothelial cell proliferation [85]. This observation parallels our observations with calretinin and is perfectly compatible with a model where both proteins promote HGSC invasion and metastasis by promoting cancer cell adhesion. Podoplanin and calretinin therefore represent potentially interesting targets to interfere with HGSC progression.

## 5 Conclusions

Transcoelomic spread triggered by cancer cells and spheroids floating in the peritoneal fluid is the major mechanism of HGSC metastasis. According to the prevailing hypothesis, these spheroids are able to attach to the ECM at lesions in the mesothelial cell layer as a prerequisite to invading through the submesothelial basement membrane. However, our knowledge of the critical molecular events and cellular interactions is incomplete. In the present study, we show by targeted sequencing that out of 21 loci previously proposed as targets of HGSC driver mutations, only *FAT3* showed a clearly increased frequency of mutations in metastases in two patients. We conclude from these findings that (i) additional mutations in most of the known HGSC driver genes apparently are not required for the acquisition of metastatic properties, and (ii) alterations in *FAT3* may contribute to the metastatic spread in a subset of patients, consistent with its function as an atypical cadherin and its known tumor‐suppressive potential. In contrast to the genetic similarities between spheroids from ascites and metastatic lesions, we identified clear and consistent differences by transcriptomic profiling, which could be emulated by the adherence of ascites‐derived spheroids in cell culture. Besides hallmarks of EMT, the adhesion‐induced transcriptome was characterized by the upregulation of the mesothelial genes *CALB2* and *PDPD*, coding for calretinin and podoplanin, respectively. Intriguingly, high *CALB2* or *PDPN* expression was strongly associated with a poor clinical outcome, and calretinin expression was observed at the invasive tumor edges of HGSC metastases. Furthermore, silencing of *CALB2* inhibited the adhesion of detached HGSC cells to a collagen type I matrix. We therefore conclude that mesothelial functions acquired by HGSC cells may be instrumental in their adhesion to the ECM of serous membranes lining the peritoneal organs and therefore contribute to HGSC metastasis.

## Conflict of interest

The authors declare no conflict of interest.

## Author contributions

RM, SR, and AB designed the study and oversaw the project. KH and AB contributed the targeted sequencing data. KO performed the experiments in Figs 3C,D,7,8, and 9. CB and MN carried out the immunohistochemical analyses of paraffin sections. SR established and characterized OCMI cell cultures from tumor cells from patients. AN and TS performed next‐generation sequencing for RNA‐Seq. EP carried out immunoblotting experiments. JMJ and UW provided clinical samples and analyzed clinical data. FF and RM performed bioinformatic and biostatistical analyses. RM wrote the paper. All authors reviewed the results and approved the final version of the manuscript.

## Supporting information


**Fig S1.** Immunostaining of p53 in two metastases from the same patient with a stabilizing C277G mutation.
**Fig S2.** Immunoblot analysis of p53 protein and its target p21 in OCMI cells.
**Fig S3.** Expression of attachment‐regulated genes in paired samples of cell spheroids from HGSC ascites and solid tumor tissue.
**Fig S4.** Immunohistochemical staining of the metastasis in Fig. 3A for EPCAM, calretinin, smooth muscle actin (SMA) and TWIST.
**Fig S5.** Immunohistochemical staining of tumor cell spheroids from HGSC ascites for the mesenchymal marker TWIST.
**Fig S6.** Downregulation of *CALB2* mRNA levels by siRNA‐mediated interference.
**Fig S7.** Flow‐cytometry‐based analysis of calretinin protein expression.

**Table S1.** Patients and tumor samples for targeted sequencing.
**Table S2.** Genes analyzed for genetic alterations by targeted sequencing.
**Table S3.** Genetic alterations identified in patient samples.
**Table S4.** Genes upregulated in adherent ascites‐derived OCMI cells versus spheroids from ascites (FC > 10).
**Table S5.** Genes downregulated in adherent ascites‐derived OCMI cells versus spheroids from ascites (FC > 50).
**Table S6.** Panther gene list of ECM‐associated genes upregulated in adherent OCMI cells.
**Table S7.** Genes highly expressed in mesothelial cells (TPM>250) versus adherent OCMI cells (FC ≥ 5).
