# Supplementary material for: Upregulation of mesothelial genes in ovarian carcinoma cells is associated with an unfavorable clinical outcome and the promotion of cancer cell adhesion
Source: Mol Oncol. 2020 Jun 25;14(9):2142–62. doi: 10.1002/1878-0261.12749 (PMC7463315; doi:10.1002/1878-0261.12749)
Supplement: Supplementary file 1 — Fig S1. Immunostaining of p53 in two metastases from the same patient with a stabilizing C277G mutation. Fig S2. Immunoblot analysis of p53 protein and its target p21 in OCMI cells. Fig S3. Expression of attachment‐regulated genes in paired samples of cell spheroids from HGSC ascites and solid tumor tissue. Fig S4. Immunohistochemical staining of the metastasis in Fig. 3A for EPCAM, calretinin, smooth muscle actin (SMA) and TWIST. Fig S5. Immunohistochemical staining of tumor cell spheroids from HGSC ascites for the mesenchymal marker TWIST. Fig S6. Downregulation of CALB2 mRNA levels by siRNA‐mediated interference. Fig S7. Flow‐cytometry‐based analysis of calretinin protein expression. [file MOL2-14-2142-s001.docx]

**Supplemental Figures**

**Fig. S1: Immunostaining of p53 in two metastases from the same patient with a stabilizing C277G mutation.** Top: OC122 metastasis with two distinct areas of p53-negative and p53-positive tumor cells. Bottom: OC122 metastasis consisting of mostly p53-positive tumor cells. Scale bar: 100 µm.

**Fig. S2: Immunoblot analysis of p53 protein and its target p21 in OCMI cells.** The indicated OCMI cells were treated with 10 µM Nutlin-3a (Sigma) or 10 µM DMSO (solvent control) for 24 hrs. Wild-type, but not mutated p53 is degraded by MDM2. Therefore, the MDM2 inhibitor Nutlin-3a prevents degradation of, and leads to high steady-state levels of, wild-type p53 but does not affect mutated p53 (Timofeev et al., 2019).

**Fig. S3: Expression of attachment-regulated genes in paired samples of cell spheroids from HGSC ascites and solid tumor tissue.** Published transcriptome data (Patch et al., 2015) were analyzed for expression of the top 15 genes upregulated upon attachment of cultured OCMI cells (Table S4; Fig. 2B) was analyzed in solid tumor tissue relative to tumor cell spheroids from the same patients. To estimate the extent of host cells we included the immune cell markers *CD4, FCGR3A (CD16), CD14* and *CD163* as well as the mesothelial markers *BEST1* and *HSPG2*. *EIF1AD* is a non-regulated standard gene. Boxplots show medians (horizontal line in boxes), upper and lower quartiles (boxes) and range (whiskers). Significance was determined by unpaired, two-tailed t test *: p<0.05, **: p<0.01, ***: p<0.001, ****: p<0.0001.

**Fig. S4: Immunohistochemical staining of the metastasis in Fig. 3A for EPCAM, calretinin, smooth muscle actin (SMA) and TWIST.** CT: connective tissue. Tumor cells are positive for all four markers. (A) EPCAM staining is considerably weaker than in spheroids (compare to Fig. 4). (B) Calretinin expression is found only at the tumor margin. (C) SMA staining is particularly strong in the adjacent connective tissue. (D) Nuclear TWIST is observed in all tumor cells at varying degrees. Scale bar: 300 µm.

**Fig. S5: Immunohistochemical staining of tumor cell spheroids from HGSC ascites for the mesenchymal marker TWIST.** The pictures show two representative examples of nuclear TWIST expression in a subset of tumor cells. Scale bar: 50 µm


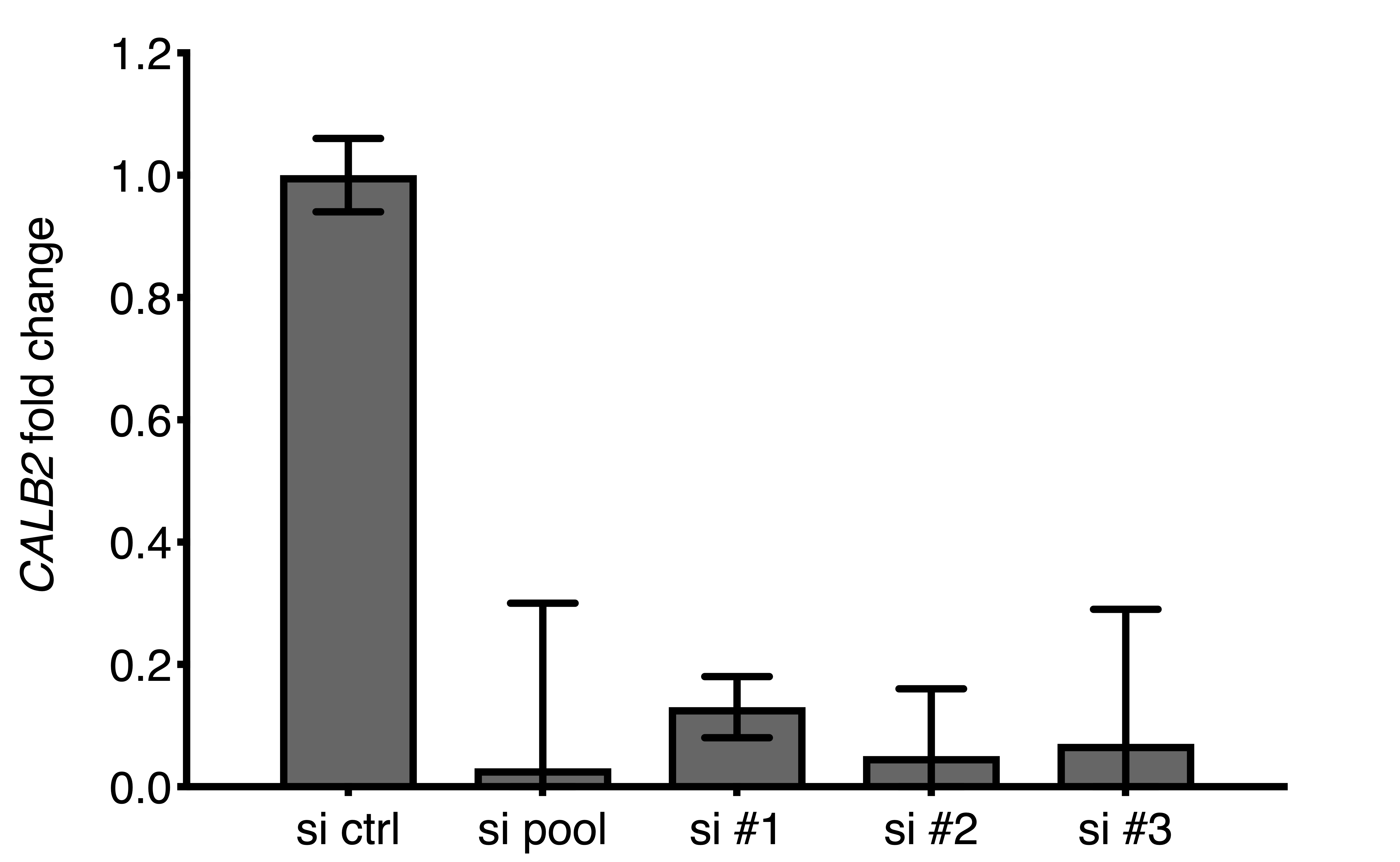


**Fig. S6: Downregulation of *CALB2* mRNA levels by siRNA-mediated interference.** OCMI137 cells were transfected with 3 different CALB2-siRNAs (si #1-3), a pool of all 3 CALB2-siRNAs and an universal siRNA negative control (si ctrl). CALB2 mRNA was quantified 96 hrs after transfection by qRT-PCR (see Materials and Methods for details). Results of technical triplicates are displayed relative to siRNA negative control. Error bars indicate the standard deviation.

**Figure S7: Flow-cytometry-based analysis of calretinin protein expression.** Percentage of calretinin-positive cells was determined for OCMI137 cells after transfection with control-siRNA or CALB2-siRNA as in Figure S4. The data were derived from 3 independent experiments. Error bars: standard deviation; ****p<0.0001 by t test.
